# Enhanced Classification of Interstitial Lung Disease Patterns in HRCT Images Using Differential Lacunarity

**DOI:** 10.1155/2015/672520

**Published:** 2015-12-22

**Authors:** Verónica Vasconcelos, João Barroso, Luis Marques, José Silvestre Silva

**Affiliations:** ^1^INESC TEC, Campus da FEUP, Rua Dr. Roberto Frias, 4200-465 Porto, Portugal; ^2^Coimbra Institute of Engineering, Polytechnic Institute of Coimbra, Rua Pedro Nunes, Quinta da Nora, 3030-199 Coimbra, Portugal; ^3^School of Science and Technology, University of Trás-os-Montes e Alto Douro, Apartado 1013, 5001-801 Vila Real, Portugal; ^4^Military Academy Research Center, Avenida Conde Castro Guimarães, 2720-113 Amadora, Portugal

## Abstract

The analysis and interpretation of high-resolution computed tomography (HRCT) images of the chest in the presence of interstitial lung disease (ILD) is a time-consuming task which requires experience. In this paper, a computer-aided diagnosis (CAD) scheme is proposed to assist radiologists in the differentiation of lung patterns associated with ILD and healthy lung parenchyma. Regions of interest were described by a set of texture attributes extracted using differential lacunarity (DLac) and classical methods of statistical texture analysis. The proposed strategy to compute DLac allowed a multiscale texture analysis, while maintaining sensitivity to small details. Support Vector Machines were employed to distinguish between lung patterns. Training and model selection were performed over a stratified 10-fold cross-validation (CV). Dimensional reduction was made based on stepwise regression (*F*-test, *p* value < 0.01) during CV. An accuracy of 95.8 ± 2.2% in the differentiation of normal lung pattern from ILD patterns and an overall accuracy of 94.5 ± 2.1% in a multiclass scenario revealed the potential of the proposed CAD in clinical practice. Experimental results showed that the performance of the CAD was improved by combining multiscale DLac with classical statistical texture analysis.

## 1. Introduction

Interstitial lung disease (ILD) is a common name for a heterogeneous group of complex disorders affecting lung parenchyma. The ILD affects similar lung regions and has identical clinical, radiological, and functional tests which hinder the differential diagnosis. However, ILD subtypes have different prognoses and treatments, so a correct diagnosis is essential [1]. High-resolution computed tomography (HRCT) imaging of the chest can offer such good image quality that it has become essential in the detection, diagnosis, and follow-up of ILD [2]. HRCT images of patients affected with ILD have specific patterns whose distribution and visual content analysis is particularly relevant in elaborating an accurate diagnosis [3].

Multidetector row computed tomography (CT) scanners generate a huge volume of data that must be visually examined by radiologists. This task is very time-consuming and requires experience, especially in the presence of ILD. Computer-aided diagnosis (CAD) for ILD is seen as a necessary tool to reduce interobserver and intraobserver variations, as well as to improve diagnostic accuracy by assisting radiologists in the detection, characterization, and quantification of pathological regions [3–13].

**Figure 1:**
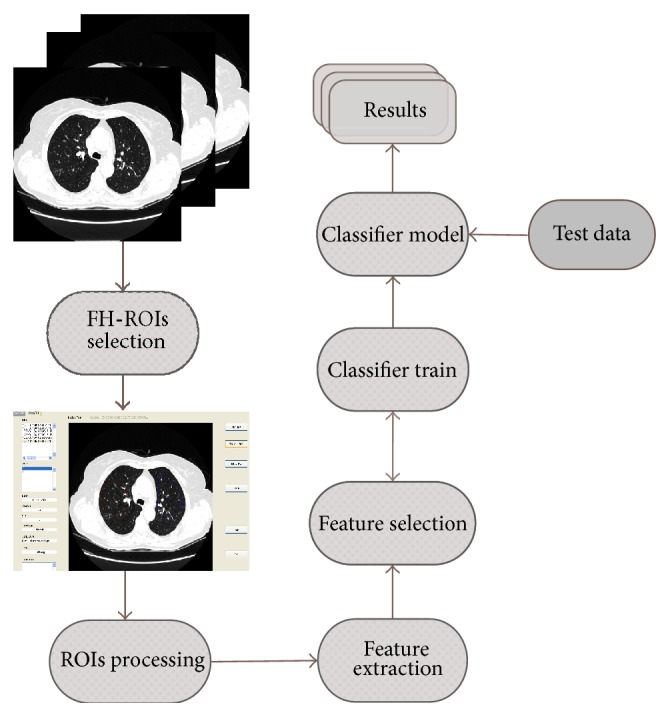
The proposed CAD scheme.

In this paper, a CAD scheme is presented allowing for a classification of regions of interest (ROIs), from HRTC images, in four classes of lung patterns: normal (NOR), ground glass (GG), honeycombing (HC), and emphysema (EMP). A scenario of binary differentiation, NOR class versus pathological class, is also considered. A generic flowchart of the proposed approach is shown in Figure 1. Classical statistical methods were used to extract and quantify texture information. The first-order (FO) analysis, the Spatial Gray Level Dependence Method (SGLDM), and the Gray Level Run-Length Method (GLRLM) allowed the estimate of statistical properties of individual pixel values and of the spatial interaction between two or more pixel values. These methods have frequently been used in texture analysis of medical images, namely, in the description of ILD patterns [4, 8–13]. Given the heterogeneity of lung parenchyma in healthy subjects or in the presence of pathologies, a multiscale texture analysis was proposed using differential lacunarity (DLac). Lacunarity has been successfully used in analyzing medical images of different organs or structures, acquired by different types of equipment. In [14, 15], fractal lacunarity analysis was applied to lumbar vertebra magnetic resonance images in order to extract relevant parameters, allowing for differentiation among three types of trabecular bone structure, from female subjects with different age and physiopathological status. In [16], lacunarity was combined with mean fractal dimension to differentiate between aggressive and nonaggressive malignant lung tumors, in sequence of contrast-enhanced CT images. An 83.3% accuracy can be valuable information in the choice of the appropriate treatment procedure. In [17], lacunarity analysis was applied for discriminating endoscopic images, obtained through a wireless capsule endoscopy technique, related to a common interstitial disease: ulceration. A promising classification accuracy of over 97% was obtained. In [18], lacunarity was applied to HRCT images of the chest to differentiate between normal and emphysematous regions of lung parenchyma. The preliminary results showed the potential of the proposed lacunarity features.

After a feature selection procedure, the obtained features were used to classify each ROI through a Support Vector Machines (SVM) algorithm. This learning algorithm has its origin in statistical learning theory and structural risk minimization [19, 20]. It emerged as an efficient technique for solving classification problems. A comparative study between SVM and other popular classifiers was performed by Meyer et al. [21]. The results highlight that SVM classifiers are among the best. In [5], five common classifiers were compared according to their ability to differentiate six lung tissue patterns in HRCT images. The results showed that SVM provides the best trade-off between the error rate and the capacity for generalization, an important aspect to take into consideration given the diversity of pulmonary patterns.

## 2. Materials and Methods

### 2.1. Texture Analysis

Texture is a major component in the interpretation of HRTC images in the presence of ILD. The most difficult aspect of texture analysis is to define a set of meaningful features that describe the texture associated with different lung patterns. Each ROI of *M* × *N* pixels was represented by a set of *m* features extracted using the methods described in the sections below.

#### 2.1.1. First-Order Statistics Analysis

The CT attenuation of each ROI was described through FO statistical features extracted from ROI normalized histogram. Considering that *L* is the number of gray levels used in ROI quantization, the normalized histogram *h*(*z*_*i*_), 0 ≤ *i* < *L*, gives the probability of observing the gray level *z*_*i*_ in the ROI. From *h*(*z*_*i*_), six statistics features were computed: the mean, variance, skewness, kurtosis, energy, and entropy [22].

#### 2.1.2. Spatial Gray Level Dependence Method

The method of texture analysis proposed by Haralick et al. [23] describes the spatial dependence of gray level distribution between neighboring pixels. In the SGLDM, the second-order joint conditional probability distribution *p*(*i*, *j*∣*dx*, *dy*) can be estimated for a defined length and along a defined direction given by offsets in *x*  and  *y* direction: *dx* and *dy*. So, *p*(*i*, *j*∣*dx*, *dy*) is the probability that two pixels at a distance given by (*dx*, *dy*) have the gray levels *i* and *j*. The function *p*(*i*, *j*∣*dx*, *dy*) is defined as follows:

(1) pi,j ∣ dx,dy=∑k=1M∑q=1Nδi,ROIk,q·δj,ROIk+dx,q+dyTdx,dy,where  δi,j=1if  i=j0if  i≠j.

ROI(*k*, *q*) is the intensity at pixel (*k*, *q*) and *T*(*dx*, *dy*) is the total number of pixels pairs belonging to the ROI in the length and direction given by (*dx*, *dy*). The functions *p*(*i*, *j*∣*dx*, *dy*) can be written in matrix form Ω(*dx*, *dy*) = *p*(*i*, *j*∣*dx*, *dy*), 0 ≤ *i*, *j* < *L*, where *L* is the maximum gray level of the ROI. For each pair (*dx*, *dy*), a different matrix Ω(*dx*, *dy*) can be computed. Often, each matrix Ω(*dx*, *dy*) is calculated taking into account a given offset and its opposite, giving rise to symmetrical matrices. In this study, from each matrix, six textural measures were extracted: angular second moment, entropy, inverse difference moment, correlation, contrast, and variance [23, 24].

#### 2.1.3. Gray Level Run-Length Method

Run-length primitives were computed by the GLRLM [25]. A run-length primitive is a consecutive and collinear set of pixels with the same gray level. These primitives can be characterized by their length, direction, and gray level. Each chosen direction gives rise to a run-length matrix Ψ(*θ*) whose elements represent the number of runs with gray level intensity *a* and length *r*, along the direction *θ*:

(2) $$\begin{array}{c} \Psi \left(\;\theta \;\right)=M\left(\;a,r\;|\;\theta \;\right),\;0\leq a<L,\;\;0<r\leq N_{r}, \end{array}$$

where *L* is the number of gray levels and *N*_*r*_ is the possible maximum run-length in ROI along *θ* direction. From each run-length matrix Ψ(*θ*), eleven features were extracted, listed, and described in [24–27].

#### 2.1.4. Lacunarity Analysis

Most of the textures and natural surfaces tend to have a fractal dimension (FD) that can be seen as a measure of irregularity [28]. However, different textures and natural surfaces can share identical FD. In order to differentiate these types of fractal patterns, Mandelbrot [29] proposed lacunarity, a complementary measure of FD that describes the texture of a fractal or their deviation from translational invariance [30]. More recent studies introduced lacunarity analysis as a technique that can be used to describe general spatial patterns, regardless of whether it is a fractal [31]. By using lacunarity, it is possible to distinguish the texture of spatial patterns through the analysis of their distribution gap sizes, at different scales.

Due to the extensive range of gray levels used in CT images acquisition, an appropriate algorithm to calculate lacunarity is that proposed in [32], called DLac. It is based on the gliding box [33] and the differential box counting algorithms [34].

**Figure 2:**
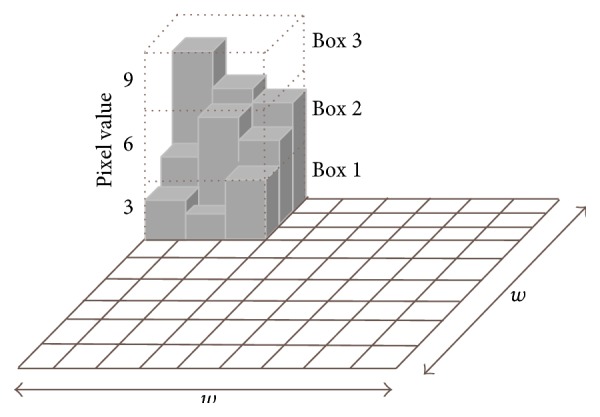
Differential box counting algorithm. A moving window of 9 × 9 pixels and a gliding box of 3 × 3 pixels are used to compute the box mass. A column of 3 cubic boxes is generated. The differential height of the column is *n* (1, 1) = 3 − 1 − 1 = 1.

According to DLac algorithm, the ROI is divided into overlapped windows of size *w* × *w* pixels, which scans the entire ROI, and a box of size *r* × *r* pixels, which scans each window (*r* < *w*). The box is placed on the left corner of the window *w* and a column of accumulated cubes of size *r* × *r* × *r* is used to cover the ROI intensity surface in the box place (Figure 2). A sequential number is assigned to each cubic box, from bottom to top. Considering that the maximum and minimum pixel values lie in the cubic box *v* and *u*, respectively, the differential height of the column is given by *n*(*i*, *j*) = *v* − *u* − 1, where (*i*, *j*) is the box position. The box mass *M* of the window *w*, at specific coordinates, is obtained gliding the box inside the entire window *w*:

(3) $$\begin{array}{c} M=\underset{i,j}{\sum }n\left(\;i,j\;\right). \end{array}$$

Considering *n*(*M*, *r*), the number of windows *w* with box mass *M* calculated through a box *r*, the respective probability function *Q*(*M*, *r*) is obtained by dividing *n*(*M*, *r*) by the total number of windows. The DLac of the ROI for a box *r*, given a window *w*, is defined as follows:

(4) $$\begin{array}{c} \Lambda \left(\;r\;\right)=\frac{\sum_{M}M^{2}Q\left(M,r\right)}{{\left[\sum_{M}MQ\left(M,r\right)\right]}^{2}}. \end{array}$$

### 2.2. Feature Selection

After performing feature extraction, it is important to proceed with the selection of the most informative features. The resulting set of optimal features improves the classifier performance, while providing a reduction of the general data, as well as a better understanding of the data. The feature selection methods can be divided into two main groups: the filter methods and the wrapper methods. In the filter methods, the features are ordered based on a relevance index. In the wrapper methods, the process of feature selection involves the predictor. In these methods, subsets of features are scored during the learning machine training according to their predictive power [35].

In this work, the reduction of dimensionality was performed using the filter method stepwise regression [36]. In this systematic method, terms are added or removed from the multilinear model based on their statistically significant *p* value of *F*-statistics. The method begins with an initial model to which terms that have *p* values less than an entrance tolerance are added, step by step, and the model terms with *p* values greater than an exit tolerance are removed from the model.

### 2.3. Support Vector Machines

The reduced number of parameters that need to be tuned as well as the good trade-off between the error rate and the capability of generalization of SVM classifier algorithm was decisive for its choice in the classification of lung patterns [5, 21].

The SVM strategy, known as the kernel trick, is to map the input data space into a higher dimension feature space, via a nonlinear function kernel *Ф* : *ℜ* ^*m*^ → *ℑ*, where separability between classes is improved. The distance between the nearest points of the two classes (*margin*) is maximized, creating an optimal separating hyperplane (OSH).

Considering the training data {**x**_*i*_, *y*_*i*_}, *i* = 1,…, *l*, **x**_*i*_ ∈ *ℜ* ^*m*^, *y*_*i*_ ∈ {+1, −1}, each instance **x**_*i*_ is characterized by a vector of *m* features (or attributes) and is associated with a class +1 or −1. The SVM machine learning solves the following quadratic optimization problem:

(5) minw,b,ξ 12w2+C∑i=1lξisubject  to yiw·Фxi+b≥1−ξi ξi≥0,  i=1,…,l,

where **w** is a normal vector to OSH and *b* is the bias. In* hard margin* SVM all the examples have to stay outside the margin and be well classified. However, in real datasets, it is necessary to deal with outliers that can be inside the margin or on the wrong side of the classification boundary. The solution proposed in [37], as the* soft margin* SVM, is to introduce constraint slack variables *ξ*_*i*_ in the optimization problem. Ideally, these variables should be zero or have small values. So, to minimize the contribution of the slack variables, a penalty term *C* is added to the objective function (5). This parameter is a trade-off between the maximization of the margin and the minimization of training errors. For a test example **x**, the decision function is given by

(6) $$\begin{array}{c} f\left(\;x\;\right)=\mathrm{sgn}\left(\;w\cdot Ф\left(\;x\;\right)+b\;\right). \end{array}$$

There are several functions kernels *K*(**x**, **x**_*i*_) = Φ(**x**) · Φ(**x**_*i*_) which can be selected to solve nonlinear problems. In this work, the Gaussian Radial Basis Function (RBF) was used: *K*(**x**, **y**) = exp(−‖**x** − **y**‖^2^)/(2*σ* ^2^). This function only has one parameter (*σ*) that has to be tuned during the classifier training and model selection.

As the standard SVM is a binary classifier, several methods were developed to extend SVM to an *n*-class problem. Typically, these methods are based on combinations of binary classifiers such as one-versus-all and one-versus-one. In the one-versus-all approach, *n* binary classifiers are trained. For example, the model of the *n*th classifier is trained using the training instances of the *n*-class as positive and all the instances of the other classes as negative. To classify a new instance, all the *n* classifiers are run on this instance. The assigned class corresponds to the classifier which returns the largest distance from the separating hyperplane. In the one-versus-one approach *n*(*n* − 1)/2 classifiers are trained in a pairwise methodology, where each takes one class as positive and the other class as negative. To classify a new example, each classifier is run and a count is assigned to each class selected by the classifier. The new instance is classified as belonging to the class which obtains the greatest number of wins, such as in a winner-takes-all voting scheme [38].

### 2.4. Dataset

The dataset *𝒟* used in this work was acquired in Radiology Department of Coimbra Hospital and Universitary Centre, Coimbra, Portugal. It contains examples of representative regions associated with GG, HC, EMP, and NOR lung patterns, obtained from the daily practice of the hospital. The examples were acquired from subjects that agreed with the use of their images for research purposes by a written consent.

A user friendly software was developed to visualize CT exams, to outline freehand ROIs (FH-ROIs), and to label and to characterize each FH-ROI [39]. HRCT scans were acquired using multidetector row CT LightSpeed VCT 64, from General Electric Healthcare, with an average voxel size of 0.7 × 0.7 × 1.3 mm^3^, without contrast agent. Each image was stored in a matrix of 512 × 512 pixels, with 16-bit gray level, using DICOM standard. Each image was displayed using a lung window with a centre in −700 Hounsfield Units (HU) and a width of 1500 HU. From CT images of 57 subjects (#29 female; #28 male) with an average age of 61 ± 16 years, radiologists outlined FH-ROIs from patients in different stages of disease.

**Figure 3:**
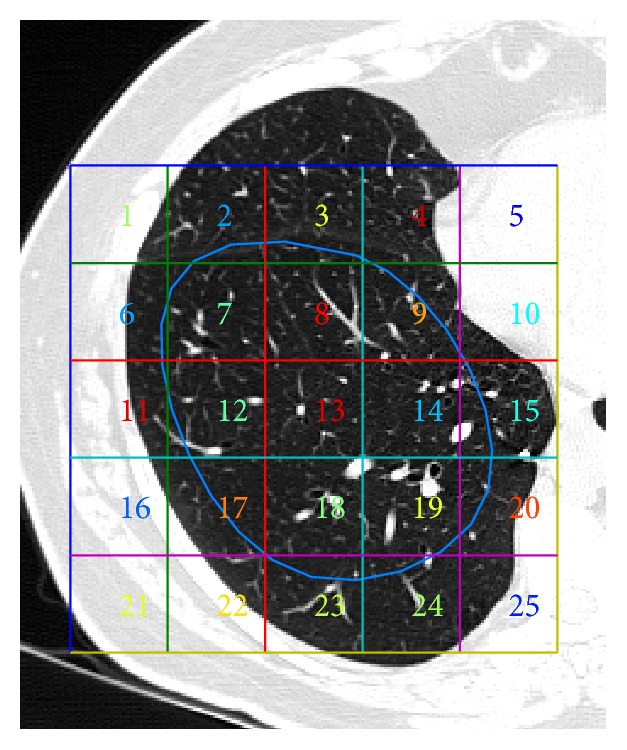
Example of FH-ROI and the grid that allows the extraction of ROIs. Only ROIs of one hundred percent  inside FH-ROI boundary were kept.

The area and shape of each FH-ROI depend on the size and localization of the lung patterns. No more than one FH-ROI was selected from each side of the lungs. The lung region of each FH-ROI was sampled and covered with contiguous, nonoverlapping ROIs of 40 × 40 pixels [24]. Each FH-ROI was sequentially numbered and each ROI holds the reference of the FH-ROI from where it was extracted. For example, the FH-ROI*x*S*y* corresponds to ROI* y* extracted from FH-ROI* x*. Only the ROIs one hundred percent inside the FH-ROI boundary were considered in the train and test of the classifier; all the other ROIs were discarded. For example, in Figure 3 only the ROIs 8, 13, 14, and 18 respect the constraint.  Table 1 resumes the dataset used to train and evaluate the proposed CAD system.

### 2.5. Model Selection and Performance Evaluation

The dataset *𝒟* (#1261 ROIs) was divided into a training set and a testing set in a proportion of 2/3–1/3, respectively. The samples were randomly selected using a holdout strategy with stratification, which ensures mutually exclusive partitions where the class proportions are roughly the same as those in the original dataset *𝒟* [40]. The holdout procedure was based on FH-ROIs in order to ensure that ROIs extracted from the same FH-ROI are placed on only one of the sets, train or test.

During SVM training, a search was carried out to find optimal parameters to create the classifier model. In the case of the selected RBF kernel function, the parameters that have to be tuned were *σ* and *C*, the regularization parameter that corresponds to a penalty over the training errors. The search for the optimal parameters was done using a grid search methodology in the hyperparameter space. So, for every point of the hyperparameter space, a *k*-fold stratified cross-validation (CV) was performed, with *k* = 10 [40, 41]. The train set was randomly split into *k* mutually exclusive folds *F*_1_, *F*_2_,…, *F*_*k*_, with approximately the same proportion of each class as in *𝒟*. During CV, the classifier was trained and tested *k* times. In each iteration, it was trained on *k* − 1 folds and tested in the remaining fold *F*_*t*_, with *t* ∈ {1,2,…, *k*}. The average of the *k*-fold accuracy corresponds to CV accuracy. To avoid the model overfitting, the feature selection procedure was included in CV loop [35]. The parameters and features that allow the best CV accuracy were selected and a fine grid search was carried out around the selected parameters, for refinement. The final classifier model was built using all the training set, the selected features, and the optimal parameters previously found. The obtained model was evaluated in the test set, which was not used during classifier training.

The performance evaluation of the classifier was performed based on a contingency table, as exemplified in Table 2 for *n*-class. Each matrix element has two indices; the first one corresponds to actual disease, while the second one corresponds to predicted disease. The elements of the main diagonal have equal indices representing correct classifications. All the other elements of the matrix correspond to incorrect classifications. For example, *a*_31_ means that a patient with disease 3 was misclassified as having disease 1. In the case of a binary classification, there are only normal and pathological classes, in a one-versus-all configuration.

After classification, the contingency table was filled with the obtained results. From these values, it is possible to compute a set of metrics allowing for the evaluation of the classifier performance. A common performance evaluation is overall accuracy, which measures the proportion of correctly classified instances for all the classes. Sensitivity of class *i* measures the fraction of actual positive instances of that class that are correctly classified, while precision measures the correctness of the predictions for class *i*. Specificity of class *i* measures the fraction of actual negative instances of class *i* that are correctly classified. These metrics can be computed by the following expressions [42]:

(7) Sensitivityi=aii∑jaij,Precisioni=aii∑jaji,Specificityi=1−∑jaij−∑j≠iaji1−∑jaij,Overall  Accuracy=∑i=jaij∑i∑jaij.

### 2.6. Feature Settings

Each ROI was characterized by a feature vector extracted using FO, SGLDM, GLRLM, and DLac.

The ROIs were quantized to 32 gray levels before the extraction of FO, SGLDM, and GLRLM features. The minimum and maximum HU values were calculated for all ROIs of *𝒟* and each ROI was quantized according to this range. In SGLDM and GLRLM, the directions *θ* = {0°, 45°, 90°, 135°} were considered. In GLDM, the distance between neighboring pixels was *d* = 1, in the four directions.

A multiscale approach was required due to the high variability of the appearance of lung patterns, even for the same pattern. The selected approach to calculate DLac allows a texture analysis at different scales by changing the value of *w*, for a box size *r*. The size of the window *w* determines the coarseness of the scale. The size of *r* should be relatively small in order to maintain sensitivity to small details present in the neighboring areas. Equation (8) illustrates the proposed approach to extract DLac features:

(8) $$\begin{array}{c} \Lambda \left(\;w,r\;\right)=\frac{\sum_{M}M^{2}Q\left(M,r,w\right)}{{\left[\sum_{M}MQ\left(M,r,w\right)\right]}^{2}}. \end{array}$$

DLac was computed for every box-window combination, subject to the condition *r* < *w*, in order to evaluate the DLac features that better differentiate the lung patterns. A DLac curve Λ(*w*, *r* = const) can be obtained by keeping the size of the gliding box *r* constant and by changing the size of the window *w*. To assure a common referential, DLac values were normalized in relation to the DLac value corresponding to the smallest window *w*: Λ(*w*_min⁡_, *r* = const) [43]. In order to take advantage of the extensive scale used in CT images, the curves of normalized DLac were computed using Hounsfield scale [−1000 UH; +1000 UH].

## 3. Results and Discussion

Two scenarios were considered in order to evaluate the potential of the proposed CAD and the importance of DLac features in the CAD performance improvement. In the first approach, the differentiation between normal and ILD patterns was considered. The next step was the differentiation of the four classes. In both cases, the feature vector was obtained using two different sets. Set 1 includes the features from FO + SGLDM + GLRLM. Set 2 also englobes DLac features.

The DLac features were extracted from DLac normalized curves. Various experiments have been conducted computing DLac curves for every box-window size for *r* = [2–34] pixels and *w* = [3–35] pixels. The ability to differentiate the four classes was evaluated. The best results were obtained for DLac normalized curves for *r* = 4 pixels and *w* = [5–35] pixels.

**Figure 4:**
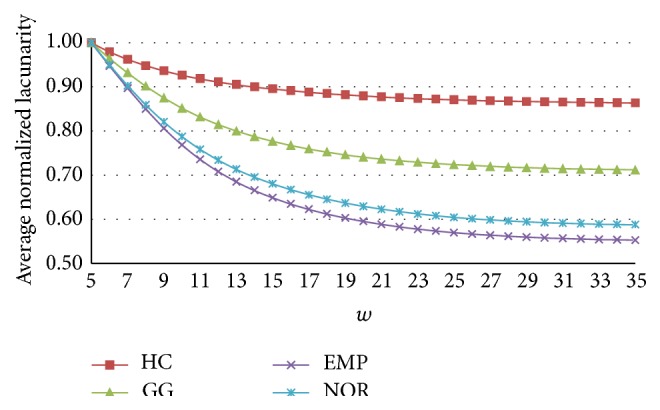
Averaged normalized DLac curves obtained for *r* = 4 pixels and *w* = [5–35] pixels.

Figure 4 shows the average of normalized DLac curves for patterns of all the dataset *𝒟*. The results show that the DLac normalized curves are able to distinguish between lung patterns, being suitable to extract informative features.

**Figure 5:**
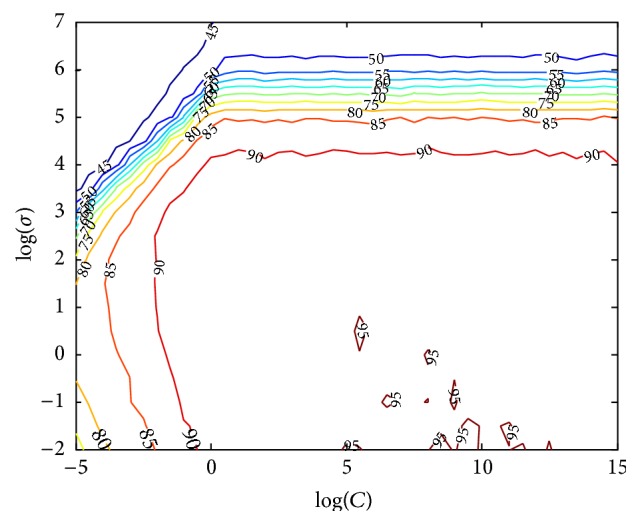
Example of 10-fold CV accuracy (%) obtained along the hyperparameter space for finding optimal parameters (*C*, *σ*). Results were obtained using Set 1, for the binary classification scenario.

The multiclass classification was performed using one-versus-one implementation [44]. In the case of the RBF kernel function, the parameters optimization was performed for the pair (*C*, *σ*). First, the parameters were evaluated using a coarse grid for *C* = 2^−5^, 2^−4.5^,…, 2^15^ and *σ* = 2^−2^, 2^−1.5^,…, 2^7^.  Figure 5 depicts a graphic of contours of CV accuracy, obtained over a 10-fold CV using features of Set 1, for the binary classification scenario. A heuristic analysis of these curves provides a clear understanding of the influence of the parameters in the classifier performance, as well as clues to reduce search space. The results showed the importance of fine tuning the SVM parameters during the classifier training phase to achieve an optimized model. After some experiments, the search grid was reduced to *C* = 2^3^, 2^3.5^,…, 2^13^ and *σ* = 2^−2^, 2^−1.5^,…, 2^1^. For every coordinate of the hyperparameter space, a *k*-fold CV was performed, with *k* = 10. In each of the *k* iterations feature selection was performed (*F*-test, *p* value < 0.01) in the *k* − 1 training folds. If the coordinates (2^*c*^, 2^*σ*^) generate the best CV accuracy, a finer search was performed around these values with a step of 0.25 upward and downward.

After the classifier training, the selected model was evaluated in the testing set. The training and testing of the classifier were repeated over fifty iterations. So, the training and evaluation of the classifier were performed in fifty different sets. The presented metrics are the average of the results obtained over all the iterations.

In the binary classification scenario, the ROIs with normal pattern (#253) were considered as negative instances and the other ones as positive instances (#1008). In Table 3, the mean and standard deviation (SD) of overall accuracy, sensitivity, precision, and specificity are shown. The classifier performance obtained using features of Set 1 was 94.4 ± 2.0% for accuracy, 96.7 ± 1.2% for sensitivity, 96.0 ± 2.1% for precision, and 84.8 ± 8.6% for specificity. Using Set 2, the results increased to 95.8 ± 2.2% for accuracy, 97.9 ± 1.1% for sensitivity, 96.9 ± 2.1% for precision, and 88.1 ± 8.0% for specificity. High sensitivity and small SD values showed that the proposed CAD has the ability to signal the presence of abnormal patterns using both sets of features; that is, the number of false negatives is low. The integration of DLac features has primarily increased the specificity value, 3.3% on average, reducing the number of false positives. However, the SD value remains high (8.0). The correct classification of NOR class instances is not easy due the high variability of healthy lung tissue.

The classifier performance in the multiclass scenario was also improved using Set 2 (Tables 4 and 5). The overall accuracy increased from 91.9 ± 1.9% to 94.5 ± 2.1%. Moreover, the class-specific metrics for NOR, GG, and HC improved in a higher or lower percentage. For class EMP sensitivity slightly increased from 96.9% to 97.3%, the precision and the specificity maintained excellent values of 99.9%. Sensitivity of NOR class was the metric that most improved with a mean increase of about 5.3%, changing from 87.2% to 92.5%. In the case of NOR class these results mean that class-specific false negatives decreased; that is, the number of instances of NOR class that were categorized as pathological instances is smaller. In a clinical environment, this means that fewer patients are subjected to the stress of unnecessary additional medical exams. NOR class-specific precision and specificity improved from 89.6% to 92.3% and from 97.3% to 97.9%, respectively. These results mean that the number of false positives for NOR class, that is, pathological instances classified as normal, decreased with Set 2. This type of misclassification has a serious meaning; that is, the CAD system does not signal the presence of a pathological pattern. The number of false negatives and false positives of GG and HC classes also decreased with the presence of DLac features increasing the correct classification of GG and HC instances. SD decreases in all metrics and classes, except for sensitivity of EMP class. So, the DLac features also improved the classifier stability.

**Figure 6:**
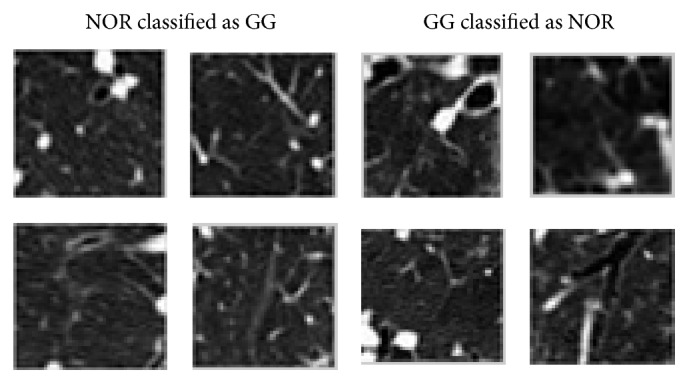
Examples of misclassified ROIs between GG and NOR classes.

The highest percentage of misclassified examples occurred among NOR and GG classes. Almost fifty percent (47.8%) of all the classification errors were due to incorrect classifications between these two classes.  Figure 6 illustrates some random examples of normal ROIs that were classified as GG, on left column, and examples of ROIs with GG pattern that were classified as NOR, on right column. Although GG opacities are characterized by areas of increased attenuation, sometimes they are not dense enough to “hide” the bronchovascular markings, especially in the initial phases of ILD diseases, associated with the presence of GG patterns.

## 4. Conclusions

A CAD scheme applied to HRCT images of the chest was proposed for the classification of healthy lung regions and with the presence of ILD. A texture analysis was performed to describe the lung patterns in study. Texture information of each ROI was represented by features extracted using a multiscale DLac approach combined with features obtained by classical statistical texture analysis methods. Feature selection and SVM training was performed over a 10-fold stratified CV. The performance evaluation of the classifier model was assessed using an independent test set.

Experimental results showed that DLac features improve the performance of the proposed CAD system in both suggested scenarios: normal versus pathological and multiclass. In this case, the number of false negatives and false positives of NOR class decreased, as well as the misclassification between instances of pathological classes. Differentiating the normal pattern from pathological patterns, the classifier accuracy improved with an average of 1.4% when DLac features were considered, resulting in a correct classification of 95.8 ± 2.2% of all instances. In the multiclass scenario the overall accuracy was improved from 91.9 ± 1.9% to 94.5 ± 2.1% due to the presence of DLac features. The performance of the proposed CAD highlights the good discriminatory properties of extracted DLac features, making it suitable to integrate clinical applications for the classification of patterns associated with ILD.

## Figures and Tables

**Table 1:** Dataset used to train and evaluate the CAD system.

Class	Normal	Ground glass	Honeycombing	Emphysema
Visual aspect	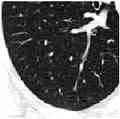	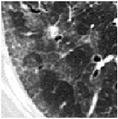	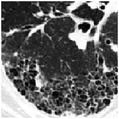	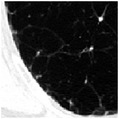

# of patients	16	20	7	14

# of freehand ROIs	87	166	72	92

# of ROIs	253	396	217	395

**Table 2:** Generic contingency table for *n*-class scenario.

Actual	Predicted		
	Class 1	⋯	Class *n*
Class 1	*a*_11_	⋯	*a*_1*n*_
⋮	⋮	*⋱*	⋮
Class *n*	*a*_*n*1_	⋯	*a*_*nn*_

**Table 3:** Mean (SD) accuracy, sensitivity, precision, and specificity using Set 1 and Set 2, for the binary classification (normal versus pathologic). Values in percentage, obtained for 50 iterations.

	Set 1	Set 2
Accuracy	94.4 (2.0)	95.8 (2.2)
Sensitivity	96.7 (1.2)	97.9 (1.1)
Precision	96.0 (2.1)	96.9 (2.1)
Specificity	84.8 (8.6)	88.1 (8.0)

**Table 4:** Mean (SD) of class-specific sensitivity, precision, and specificity using Set 1, for the multiclass classification. Values in percentage, obtained for 50 iterations.

	Classes			
	NOR	GG	HC	EMP
Sensitivity	87.2 (4.6)	92.5 (2.4)	89.4 (4.6)	96.9 (1.7)
Precision	89.6 (3.8)	84.7 (4.5)	93.5 (2.6)	99.8 (0.5)
Specificity	97.3 (1.1)	93.4 (2.3)	98.6 (4.6)	99.9 (0.2)

**Table 5:** Mean (SD) of class-specific sensitivity, precision, and specificity using Set 2, for the multiclass classification. Values in percentage, obtained for 50 iterations.

	Classes			
	NOR	GG	HC	EMP
Sensitivity	92.5 (3.8)	96.7 (3.0)	92.3 (4.0)	97.3 (1.8)
Precision	92.3 (3.4)	88.9 (3.9)	97.5 (2.5)	99.9 (0.3)
Specificity	97.9 (1.1)	95.2 (1.8)	99.5 (4.0)	99.9 (0.1)
